# Some Comments and a Criticism

**Published:** 1920-07-17

**Authors:** 


### THE DE LA RUE SCHEME OF MEDICAL SERVICE.

### Some Comments and a Criticism.

From an authoritative communication sent to us we learn that the firm of Messrs. Thomas De La Rue and Company, Limited, desires to secure for its employés the advantages of a highly efficient medical service, when, as the result of illness or accident, aid of this order becomes necessary. The proposal accepts the view that many workers with moderate incomes are not able readily to pay the ordinary fees of consulting physicians and surgeons, and yet are not of the class for which the voluntary hospitals exist. Hence such persons, in the event of serious or complicated illness, are either deprived of the opportunity of the most experienced medical advice, or are driven, against their will, to seek such advice at the hands of a charitable institution. The one way to meet this position obviously is to arrange a scale of consultation fees which may be described as reasonable in relation to the incomes of the persons immediately concerned. This is the plan which the firm has adopted, and we gather that the fees fixed have received the endorsement of certain members of the medical profession. The Company, it appears, already provides a medical adviser for the benefit of its workers, and the proposal now is that on the certificate of this gentleman any employé may be sent for advice or treatment to one or other of the physicians or surgeons who have been nominated by the firm as authoritative in the various departments of consulting practice. Whether the patient himself will have any choice in the matter does not appear, but in any event the selection is limited to the names which are announced in the printed list, and to these for all services rendered the Company will pay that proportion of the ordinary fee which corresponds to the income of the patient as this is determined by the adjusted scale. There is a further suggestion. It is that other large employers should follow the example here set and that various firms should combine to organise and conduct, on a business basis, a nursing home where the charges should be regulated according to the income of the patient.

### An Excellent Motive.

Our first word on the above scheme is to recognise the excellence of the motive which has prompted it. There can be no doubt that many persons with limited and moderate incomes are, in the event of serious illness or accident, placed in a cruel dilemma. To accept charity is naturally and properly repugnant to them, and yet they must either do this or, in the alternative, pay fees which are quite out of proportion to their resources. A tale of medical attendances and of consultation fees and nursing charges may be a cruel and crippling burden for many a middle-class household. It is true that consulting physicians and surgeons are almost invariably willing to modify their fees to meet the existing circumstances, and this is entirely in accordance with the best traditions of the profession. But the custom is liable to abuse by unscrupulous persons, and probably the most deserving are the least ready to ask for a concession which has the appearance of a favour rather than a right. Hence there must be an obvious advantage in a scheme where the patient's income is certified by a responsible authority and where, as an automatic consequence, the fee due for a professional consultation is fixed in advance. Both in its motive and in its principle, therefore, the De La Rue service deserves, in our judgment, cordial recognition.

Yet—at least, so it seems to us—the scheme has serious defects, alike from the point of view of the patient and from that of the medical profession. If there is one principle on which the profession is agreed in relation to any form of organised medical service it is that the patient shall enjoy a free choice in the selection of his adviser. But, according to the scheme now under review, no such choice will be possible. There is an official list of two or three names in medicine, in surgery, and in each of the more specialised departments, and to these the patient is restricted. Whether this list is to be circulated among the firm's employees or not we do not know, but if it is the practitioners who appear on it can hardly escape the attention of the General Medical Council. The obscure practitioner whose name is printed on the card of a Medical Benefit Society has in his day suffered penalties for touting for patients, and on any principle of equal justice the decorated denizens of Harley Street must suffer the same verdict. We are told that "the medical profession have given their whole-hearted support to this scheme," but on what authority this statement rests we do not know, and though we are in the way of hearing of such matters, no information has reached us that the proposal has been submitted to any body competent to speak on behalf either of the profession as a whole or of any considerable section of it. Nowadays proposals which aspire to professional endorsement must come out of holes and corners into the light of day, and while we recognise the excellent intentions of the De La Rue firm, we cannot say that they have been well guided by their medical advisers. What might have been an illustration and an example of the fashion in which medical help might be organised to meet the position of the middle classes appears rather as a closed corporation for the benefit and advertisement of a selected group of medical men and with the suggestion that these are more competent and more benevolent than their neighbours.

**The De La Rue Scheme of Medical Service**—(*cont.*).

Another Aspect.

A still more objectionable feature of the De La Rue proposals is a disregard of the position and values of the patient's family medical adviser. The scheme ignores him altogether. It is not on his advice, but on the certificate of "the company's medical adviser," that the machinery of consultations on the basis of graduated fees can in any individual case be brought into operation; and he, apparently, is to have neither part nor lot in the discussion. This feature alone is sufficient to show that the proposals have never been carefully considered by any responsible body of professional opinion, and it is beyond question in direct conflict both with professional judgment and professional practice. Unless it is mended there will be opposition in quarters where opposition is able to make itself effective, for practitioners, not more than patients, are ready to have consultants imposed on them by the fiat of outsiders, charged though these may be with the very best intentions.

A Serious Obstacle.

This disregard of the general practitioner will, we believe, be a serious obstacle to the success of the proposed nursing home. We are heartily in accord with the desire to organise such homes for members of the middle classes, and we have no doubt such an enterprise could be run successfully on business lines. But we are sure such institutions will be strongly and successfully opposed unless each patient is allowed the choice of his own doctor, and this in reference both to the regular attendant and to any consultant who may be desired. The notion that such homes can be run by small groups of consulting physicians and surgeons, who are ready to modify their fees in accordance with the patients' incomes, is, we are convinced, a misleading one, and an attempt to put it into practice will arouse determined opposition. It is in direct antagonism to the prevailing currents of professional opinion, and in such circumstances failure is inevitable. The physician who writes that the De La Rue proposal provides the solution of "one of the greatest of the many social problems that face us" affords apt justification of Sir Robert Morant's remark that "medical men do not think nor express themselves in a statesmanlike way"; the municipal quality of this remark helps to spoil a good case by overstating it.

The Goodwill of the Profession.

There is no need to seek far for support for our statement that no organised scheme of medical treatment which thrusts to one side the family practitioner can hope to command the goodwill of the medical profession. Presidential addresses, proposals taking origin in the Ministry of Health, medical discussions and debates are all agreed in proclaiming that constructive arrangements, whether of therapeutic or prophylactic intent, must be based upon the co-operation or even on the initiative of the general practitioner. Is it, therefore, possible to believe that a plan of large nursing homes, each offering some 250 or 300 beds, and where "eminent consulting physicians and surgeons would agree to treat privately" and at reduced fees patients sent on the certificate of the medical officer of one or other commercial firms, is likely to obtain anything like general medical support? Nor do we imagine that such arrangement would secure a high level of efficiency. Every man rests in his own order, and eminent consulting physicians and surgeons have something else to do than to engage in the "private" treatment of individual patients. They may, on occasions, contribute an element of great value, but sustained and daily supervision of patients is best left in the hands of those who have served an apprenticeship to it.

Our Appreciation.

In conclusion, we repeat our high appreciation of Messrs. De La Rue's object and motive, but we feel that on certain aspects of the scheme the firm have been ill-advised. Let them put their proposals before some responsible and representative medical organisation, and they will, we are sure, command both attention and goodwill. Whatever modifications may be suggested will be directed to secure a more satisfactory solution of the problem with which the firm, in so praiseworthy a spirit, desires to deal.

